# 4267 Noninvasive hybrid ultrasound and photoacoustic imaging for the assessment of liver fibrosis

**DOI:** 10.1017/cts.2020.338

**Published:** 2020-07-29

**Authors:** Laith Riyadh Sultan, Mrigendra Karmacharya, Julia D’Souza, Brooke Kirkham, Angela K Brice, Andrew KW Wood, Stephen Hunt, Chandra Sehgal

**Affiliations:** 1 University of Pennsylvania School of Medicine

## Abstract

OBJECTIVES/GOALS: The detection of liver fibrotic changes at an early and reversible stage is essential to prevent its progression to end-stage cirrhosis and hepatocellular carcinoma. Liver biopsy, which is the current gold standard for fibrosis assessment, is accompanied by several complications due to its invasive nature in addition to sampling errors and reader variability. In this study, we evaluate the use of quantitative parameters extracted from hybrid ultrasound and photoacoustic imaging to detect and monitor fibrotic changes in a DEN rat model. METHODS/STUDY POPULATION: Liver fibrotic changes were induced in 34 Wistar male rats by oral administration of Diethylnitrosamine (DEN) for 12 weeks. 22 rats were imaged with B-mode ultrasound at 3 different time points (baseline, 10 weeks and 13 weeks) for monitoring liver texture changes. Texture features studied included tissue echointensity (liver brightness normalized to kidney brightness) and tissue heterogeneity. 12 rats were imaged with photoacoustic imaging at 4 time points (baseline, 5 wks, 10 wks, and 13 wks) to look at changes in tissue oxygenation. Hemoglobin oxygen saturation (sO2A) and hemoglobin concentration (HbT) in the right and left lobes of the liver were measured. 8 rats were used as controls. Liver tissue samples were obtained following 13 weeks from DEN start time for METAVIR histopathology staging of fibrosis. RESULTS/ANTICIPATED RESULTS: Texture features studied showed an increase with time in DEN rats. Normalized echointensity increased from 0.28 ± 0.06 at baseline to 0.46 ± 0.10 at 10 weeks (*p* < 0.0005) and 0.53 ± 0.15 at 13 weeks in DEN rats (*p* < 0.0005). In the control rats, echointensity remained at an average of 0.25 ± 0.05 (*p* = 0.31). Tissue heterogeneity increased over time in the DEN-exposed rats from a baseline of 208.7 ± 58.3 to 344.6 ± 52.9 at 10 weeks (*p* < 0.0005) and 376.8 ± 54.9 at 13 weeks (*p* = 0.06) however it stayed constant at 225.7 ± 37.6 in control rats (*p* = 0.58). The quantitative analyses of the photoacoustic signals showed that blood oxygen saturation significantly increased with time. At 5 weeks sO2AvT increased by 53.83 % (± 0.25), and HbT by 35.31 % (± 0.07). Following 10 weeks of DEN; sO2AvT by 92.04 % (± 0.29), and HbT by 55.24 % (± 0.1). All increases were significant *p* < 0.05. In the 13th week, however, the values of all of these parameters were lower than those in the 10th week, however, the decrease was statistically insignificant. DISCUSSION/SIGNIFICANCE OF IMPACT: Quantitative features from B-mode ultrasound and photoacoustic imaging consistently increased over time corresponding to hepatic damage, inflammation and fibrosis progressed. The use of this hybrid imaging method in clinical practice can help meet the significant need for noninvasive assessment of liver fibrosis.

